# Fatal Hemorrhage Due to Aorto-Enteric Fistula: A Case Report

**DOI:** 10.7759/cureus.37620

**Published:** 2023-04-15

**Authors:** Tajah M Alaithan, Abdullah M Alaithan, Lujain M Alnasser, Ali A Alnakhli, Ahlam Alharbi

**Affiliations:** 1 General Practice, Almaarefa University, Riyadh, SAU; 2 General Practice, Al-Omran General Hospital, Al-Ahsa, SAU; 3 General Practice, King Abdulaziz University, Jeddah, SAU; 4 General Practice, Al-Haram Hospital, Medina, SAU; 5 Family Medicine, Primary Health Care, Riyadh, SAU

**Keywords:** case report, gastrointestinal bleeding, hypovolemic shock, aorto-enteric fistula, abdominal aortic aneurysm

## Abstract

The abdominal aortic aneurysm (AAA) is a vascular condition that commonly affects individuals over the age of 65, leading to complications such as rupture, thrombosis, and embolization that can result in significant morbidity and mortality. Aorto-enteric fistula (AEF), a rare but life-threatening complication of abdominal aortic aneurysms, occurs when there is communication between the aneurysm and adjacent bowel loops. A 63-year-old man presented to the emergency department (ED) with severe abdominal pain, nausea, vomiting, and dark, tarry stools. Prior to his current presentation, the patient sought medical care from several primary care centers for vague abdominal pain that was diagnosed as dyspepsia, and he was prescribed omeprazole. During the current presentation, the patient had hemodynamic instability and a diffusely tender abdomen. Subsequently, a computed tomography (CT) scan revealed an abdominal aortic aneurysm with AEF. Although the patient underwent exploratory laparotomy, he suffered cardiac arrest and ultimately died in the operating room. This case underscores the importance of early recognition and management of AEF, which is crucial for improving patient outcomes.

## Introduction

The abdominal aortic aneurysm (AAA) is a common vascular pathology that affects 1-2% of the population over the age of 65 years [1]. It can lead to significant morbidity and mortality due to complications such as rupture, thrombosis, and embolization [1]. Aorto-enteric fistula (AEF) is a rare but life-threatening complication of abdominal aortic aneurysm that occurs when there is communication between the aneurysm and adjacent bowel loops. It can manifest with a wide range of nonspecific symptoms, making the diagnosis challenging [2]. The classic triad of AEF includes abdominal pain, gastrointestinal bleeding, and a pulsatile abdominal mass. However, this triad is only present in approximately 50% of cases [2]. In some cases, AEF can cause acute, massive gastrointestinal bleeding, leading to hypovolemic shock and hemodynamic instability. Here, we present a fatal case of a patient who suffered from AEF, developed hemodynamic instability, and was preceded by vague abdominal pain for two weeks. The patient was ultimately diagnosed with AEF after a computed tomography (CT) scan in the emergency department (ED) revealed an aortic aneurysm with gas in the adjacent bowel loops. Despite prompt surgical intervention, the patient suffered cardiac arrest and expired in the operating room. This case highlights the importance of early recognition and management of AEF, particularly in patients with a history of abdominal aortic aneurysms and nonspecific symptoms.

## Case presentation

A 63-year-old man presented to the ED with severe abdominal pain, nausea, and vomiting for a duration of two weeks. The pain was initially intermittent and colicky but progressively worsened over the preceding few days. The patient sought care from several primary care centers during this time for vague abdominal pain. He had been taking omeprazole 40 mg once daily for the past 14 days with no significant improvement in symptoms. Additionally, the patient reported having dark, tarry stools and hematemesis for the preceding three days.

The patient had a past medical history of hypertension and hyperlipidemia. The patient had a 40-pack-year history of smoking but quit ten years ago. He denied any significant past surgical history or alcohol or drug abuse.

On physical examination, the patient appeared acutely ill and was hypotensive with a blood pressure of 80/50 mmHg and normal other vital signs. His abdomen was distended and diffusely tender to palpation, with guarding and rebound tenderness in the left upper quadrant. No other significant findings were noted during the rest of the physical examination.

**Figure 1:**
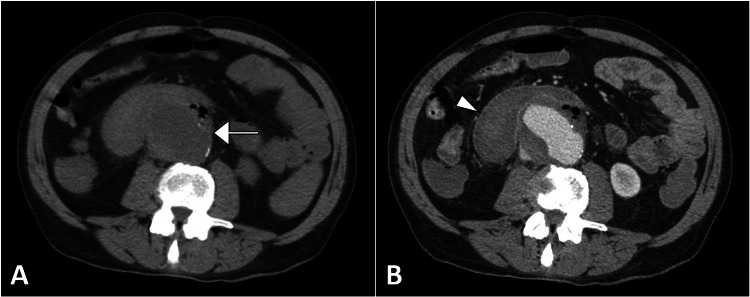
The axial CT image in the non-contrast phase (A) shows an abdominal aortic aneurysm (arrow). The contrast-enhanced image (B) highlights a faint contrast enhancement of the small bowel loop (arrowhead) adjacent to the aneurysm, confirming the presence of an aorto-enteric fistula. CT: computed tomography.

**Figure 2:**
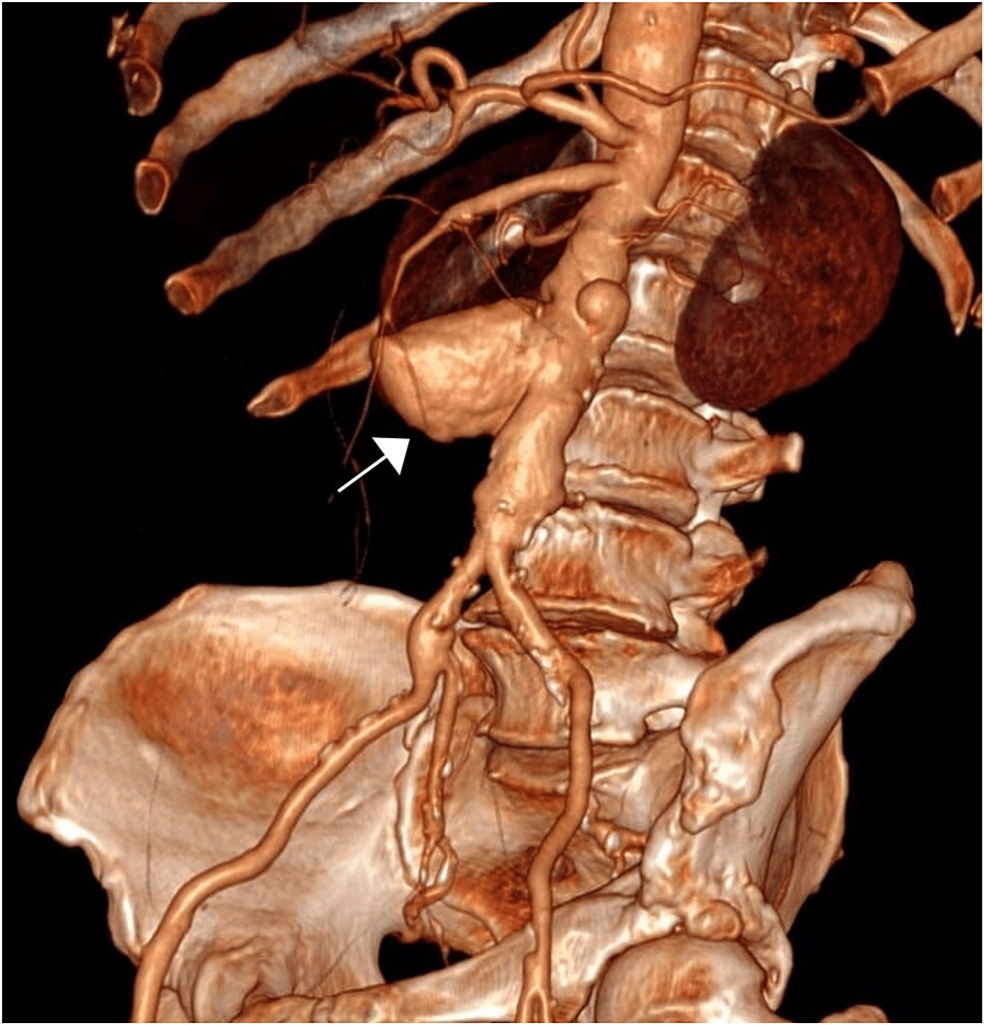
The 3D volume-rendered CT angiography reveals an aorto-enteric fistula (arrow). CT: computed tomography.

Laboratory tests showed a hemoglobin level of 8.5 g/dL and an elevated white blood cell count of 16,000/mm³. Liver function tests were within normal limits. Other laboratory results were unremarkable. A bedside ultrasound examination was not performed. A CT scan of the abdomen and pelvis was performed, which showed an abdominal aortic aneurysm measuring 6.5 cm in diameter with a focal area of intraluminal gas and extravasation of contrast into the adjacent bowel loops, suggestive of an AEF (Figures 1-2).

The patient was urgently taken to the operating room for an exploratory laparotomy and repair of the AEF. During surgery, the surgeons noted extensive intra-abdominal hemorrhage and a large defect in the anterior wall of the duodenum, adjacent to the aneurysm. However, despite the team's best efforts, the patient suffered a cardiac arrest on the operating table and was unable to be resuscitated, ultimately dying in the operating room.

## Discussion

An aorto-enteric fistula is a rare but potentially fatal complication of AAAs. The incidence of AEF is estimated to be between 0.04% and 3.3% in patients with AAAs, with the majority of cases occurring in men over the age of 65 [3]. The most common cause of AEF is the erosion of the aneurysm into the adjacent bowel, which leads to fistula formation. Other causes include infection, trauma, and iatrogenic injury. The clinical presentation of AEF is often nonspecific, with symptoms such as abdominal pain, gastrointestinal bleeding, and sepsis. The diagnosis can be challenging, and a high index of suspicion is required to make the diagnosis [4].

In this case, the patient presented with a two-week history of abdominal pain that was initially treated as dyspepsia with proton pump inhibitors. Despite medical therapy, the patient’s symptoms persisted, and he subsequently presented to the ED with hematochezia and hypotension. CT imaging revealed an AEF, which led to urgent surgical intervention. Unfortunately, the patient died on the operating table before the repair could be completed.

Diagnosing AEF is challenging, as it can manifest with a wide range of nonspecific symptoms [4]. In our case, the patient’s symptoms were initially attributed to dyspepsia, and it was not until he presented with hematochezia and hypotension that the diagnosis of AEF was considered. Delayed diagnosis and treatment of AEF can lead to high mortality rates, with reported rates ranging from 15% to 90% [3]. Therefore, it is critical to consider AEF in the differential diagnosis of patients with a history of AAA and nonspecific gastrointestinal symptoms.

Imaging modalities such as CT and magnetic resonance angiography are essential for the diagnosis of AEF. CT is the most commonly used imaging modality, with a reported sensitivity of 80-90% for the detection of AEF [4]. However, false-negative results can occur, particularly in small fistulas or those with low flow rates. Magnetic resonance angiography is an alternative imaging modality that can be used in cases where CT is contraindicated or in patients with renal insufficiency [3]. In the present case, a bedside ultrasound was not performed, and this investigation could have revealed the AAA.

The treatment of AEF is surgical, aiming to repair the fistula and resect the aneurysm. Endovascular repair may be considered in select cases, particularly in patients who are not surgical candidates [5]. However, the mortality rates for endovascular repair are higher than those for open repair, and the long-term outcomes are not well established [5].

## Conclusions

Aorto-enteric fistula is a rare but potentially life-threatening complication of abdominal aortic aneurysms. Prompt recognition and management are critical for improving the outcomes. This case underscores the importance of considering aorto-enteric fistula in the differential diagnosis of patients with risk factors for abdominal aortic aneurysm and nonspecific gastrointestinal symptoms, as prompt diagnosis and treatment can lead to improved survival. A bedside ultrasound examination is a vital investigation that could have resulted in an earlier diagnosis of this condition.
